# SIMPROT: Using an empirically determined indel distribution in simulations of protein evolution

**DOI:** 10.1186/1471-2105-6-236

**Published:** 2005-09-27

**Authors:** Andy Pang, Andrew D Smith, Paulo AS Nuin, Elisabeth RM Tillier

**Affiliations:** 1 Ontario Cancer Institute, University Health Network, Toronto, Ontario, Canada; 2 Dept. Medical Biophysics, University of Toronto, Toronto, Ontario, Canada; 3 Cold Spring Harbor Laboratory, 1 Bungtown Road, Cold Spring Harbor, NY 11724 USA

## Abstract

**Background:**

General protein evolution models help determine the baseline expectations for the evolution of sequences, and they have been extensively useful in sequence analysis and for the computer simulation of artificial sequence data sets.

**Results:**

We have developed a new method of simulating protein sequence evolution, including insertion and deletion (indel) events in addition to amino-acid substitutions. The simulation generates both the simulated sequence family and a true sequence alignment that captures the evolutionary relationships between amino acids from different sequences. Our statistical model for indel evolution is based on the empirical indel distribution determined by Qian and Goldstein. We have parameterized this distribution so that it applies to sequences diverged by varying evolutionary times and generalized it to provide flexibility in simulation conditions. Our method uses a Monte-Carlo simulation strategy, and has been implemented in a C++ program named Simprot.

**Conclusion:**

Simprot will be useful for testing methods of analysis of protein sequence families particularly alignment methods, phylogenetic tree building, detection of recombination and horizontal gene transfer, and homology detection, where knowing the true course of sequence evolution is essential.

## Background

Protein evolution has been largely modelled by considering the amino acid substitution process. There have been few statistical studies of the processes of insertion and deletion. Thorne *et al*. (1991) [2] described a theoretical parametric model that has been used to model the processes of insertion and deletion of single amino acids. The model has been extended, and others developed, to include the consideration of longer indels ([3-5]), however a model based on actual sequences may be more realistic and therefore preferable.

The study Benner, Cohen and Gonnet (1993) [6] is therefore a landmark one. The distribution of indels length was empirically determined from the alignment of conserved proteins with less than 100 PAM units of sequence divergence. This limit on the range of divergence was established in order to reduce both the redundancy of indel events counted, and the numbers of indels that resulted from independent overlapping events. In that study and in a more recent update [7], the estimate for the indel length distribution fit to a Zipfian distribution.

The study of Qian and Goldstein (2001) [1] on the other hand, derived an empirical distribution for the length of indels from a database of protein alignments sharing no more than 25% sequence identity. The distribution in that case fit a linear combination of 4 exponential functions. We call this function the Qian-Goldstein distribution and the Zipfian distributions found by [7] and [6], the Benner distributions. The Qian-Goldstein distribution is more applicable to protein sequence comparisons with long sequence divergence whereas the Benner distributions are more applicable to sequences of lower divergence. The Qian-Goldstein distribution was derived for the determination of realistic gap insertion and deletion penalties that are generally used in alignment algorithms. These affine gap penalties are used to mimic the fact that although insertions and deletions are rare events, they often involve more than one amino acid. That observation reflects the fact that some regions of protein sequence and structure are able to tolerate sections of insertion or deletion.

The evolutionary processes of mutation and subsequent natural selection determine the occurrence of substitutions, insertions and deletion. The specifics of the processes are difficult to model accurately since they are determined by many factors at all context levels (i.e. the population, the genome, the cell, and particularly the DNA and protein sequence and structure). However general protein evolution models are useful as they can help determine the baseline expectations for the evolution of sequences, and they have been extensively used for the computer simulation of artificial sequence data sets.

Two freely available programs that generate sets of sequences by Monte Carlo simulation of evolution are Seq-Gen [8,9], and Rose [10]. Seq-Gen generates sequences using a given evolutionary tree, making substitutions according to a specified model. Several models of amino acid substitution are available, including the popular PAM [11] and JTT models [12]. Additionally, Seq-Gen allows rates of evolution to vary between sites according to the gamma model developed by Yang [13]. Seq-Gen only considers substitutions and does not simulate the processes of insertion and deletion. On the other hand, the Rose program does simulate insertions and deletions, along with substitutions, but has the disadvantage of not allowing for different rates of evolution at different sites. The user determines the distribution of indel length used by Rose software. That distribution is then fixed and does not depend on evolutionary time (*i.e*. branch length in the tree); only the frequency of indels is determined by the branch length separating the ancestral and daughter sequences.

The empirically derived distribution of Qian-Goldstein [1] was obtained using a subset of structural sequence alignments corresponding to highly diverged sequences in the database. The distribution as such is limited to models of proteins corresponding to this set of circumstances. Although the Qian-Goldstein distribution is fixed with respect to evolutionary time, it has the property of being easily parameterized. We generalized the model so that it applies to proteins with variable sequence divergence and show that this generalized distribution may be comparable to the Benner distribution [7] at shorter evolutionary distances. We implemented our generalized Qian-Goldstein distribution in a new program for the simulation of protein sequences (Simprot). Like earlier programs, Simprot allows for several models of amino acid substitution, and permits gamma distributed sites rates according to the Yang [13] model. By incorporating our parameterized Qian-Goldstein model for indels, the user has flexibility to modify the distribution and obtain longer/shorter or more/less frequent insertions and deletions. Simprot is the first program to simulate protein sequence evolution with the additional capability of being able to simulate indels with a variable length distribution. Additionally, Simprot allows the protein sequence to be segmented such that the different segments can evolve with distinct sets of parameters and tree.

## Results

### Parametrization of the Qian-Goldstein indel length distribution

The empirically derived Qian-Goldstein distribution [1] (equation 8 in that paper) is given by

QG(n)=1.027×10−2e−n/0.96           +  3.031×10−3e−n/3.13           +  6.141×10−4e−n/14.3     (1)           +  2.090×10−5e−n/81.7.
 MathType@MTEF@5@5@+=feaafeart1ev1aaatCvAUfKttLearuWrP9MDH5MBPbIqV92AaeXatLxBI9gBaebbnrfifHhDYfgasaacH8akY=wiFfYdH8Gipec8Eeeu0xXdbba9frFj0=OqFfea0dXdd9vqai=hGuQ8kuc9pgc9s8qqaq=dirpe0xb9q8qiLsFr0=vr0=vr0dc8meaabaqaciGacaGaaeqabaqabeGadaaakqaabeqaauaabeqabmaaaeaacqWGrbqucqWGhbWrcqGGOaakcqWGUbGBcqGGPaqkaeaacqGH9aqpaeaacqaIXaqmcqGGUaGlcqaIWaamcqaIYaGmcqaI3aWncqGHxdaTcqaIXaqmcqaIWaamdaahaaWcbeqaaiabgkHiTiabikdaYaaakiabdwgaLnaaCaaaleqabaGaeyOeI0IaemOBa4Maei4la8IaeGimaaJaeiOla4IaeGyoaKJaeGOnaydaaaaaaOqaaiaaykW7caaMc8UaaGPaVlaaykW7caaMc8UaaGPaVlaaykW7caaMc8UaaGPaVlaaykW7caaMc8EbaeaabeWaaaqaaaqaaaqaaiabgUcaRiaaykW7caaMc8UaeG4mamJaeiOla4IaeGimaaJaeG4mamJaeGymaeJaey41aqRaeGymaeJaeGimaaZaaWbaaSqabeaacqGHsislcqaIZaWmaaGccqWGLbqzdaahaaWcbeqaaiabgkHiTiabd6gaUjabc+caViabiodaZiabc6caUiabigdaXiabiodaZaaaaaaakeaacaaMc8UaaGPaVlaaykW7caaMc8UaaGPaVlaaykW7caaMc8UaaGPaVlaaykW7caaMc8UaaGPaVxaabeqabmaaaeaaaeaaaeaacqGHRaWkcaaMc8UaaGPaVlabiAda2iabc6caUiabigdaXiabisda0iabigdaXiabgEna0kabigdaXiabicdaWmaaCaaaleqabaGaeyOeI0IaeGinaqdaaOGaemyzau2aaWbaaSqabeaacqGHsislcqWGUbGBcqGGVaWlcqaIXaqmcqaI0aancqGGUaGlcqaIZaWmaaGccaWLjaGaaCzcamaabmaabaGaeGymaedacaGLOaGaayzkaaaaaaqaaiaaykW7caaMc8UaaGPaVlaaykW7caaMc8UaaGPaVlaaykW7caaMc8UaaGPaVlaaykW7caaMc8EbaeqabeWaaaqaaaqaaaqaaiabgUcaRiaaykW7caaMc8UaeGOmaiJaeiOla4IaeGimaaJaeGyoaKJaeGimaaJaey41aqRaeGymaeJaeGimaaZaaWbaaSqabeaacqGHsislcqaI1aqnaaGccqWGLbqzdaahaaWcbeqaaiabgkHiTiabd6gaUjabc+caViabiIda4iabigdaXiabc6caUiabiEda3aaakiabc6caUaaaaaaa@C3D8@

This function describes the frequency of an indel, of any length *n *> 0, as a fraction of the average length of the protein sequence. The model accurately describes a data set of aligned sequences with less than 25% sequence identity. The total frequency of indels is estimated by ∑_*n *> 0_*QG*(*n*), which converges rapidly to 0.0238. This value is close to the observed frequency of indels (0.030) that was found by Qian and Goldstein in database they analyzed [1].

As mentioned above, the dataset used to infer Equation 1 was highly diverged, so we may assume it accurately applies to sequences of large divergence. We will therefore assume that the Qian-Goldstein applies at an evolutionary distance *c*, a parameter to which evolutionary time *t *will be scaled. This allows us to define *QG*'(*n*, *t*, *c*) = *QG*(*n*) for *n *> 0 and *t *= *c*.

However this only defines the *QG*' function at one evolutionary time point, *t *= *c*. It is necessary to define the expected distribution of observed indel lengths for all evolutionary times. The Qian-Goldstein distribution describes the *observed *length frequency after a large amount of divergence, but it does not describe the *actual *distribution of the expected rate of fixation in the population of insertion and deletion mutations (the rate of indel occurrence). This is because a single observed indel may have been the result of several actual events. Even if the length distribution for indel occurrences were known, a Markov model for the process of insertion and deletion would need to be established and used to derive the expected distribution of observed indels for any given degree of divergence. Additional empirical data is needed to derive the expected distribution of observed indel lengths scaled to other divergence times.

In the absence of additional empirical data, we must make some assumptions about the insertion and deletion processes to derive the indel length distribution for all evolutionary time.

1. We assume that the length of indels will increase with evolutionary time as larger indels are more easily tolerated and smaller ones overlap. We therefore expect that shorter indels arise over smaller divergence times and that larger indels are the result of independent but contiguous events. We design the distribution such that it is has the property that the limit as time goes to 0 for the expected frequency of all indels (>1), is also 0. This assumes that the instantaneous rate of an indel involves only a single amino acid, which is unlikely (see for example [14]). The assumption is only approximatively true even if the mutation process created only single amino acid indels because multiple mutations may be fixed by natural selection and genetic drift. The effect on the indel model will be that the lengths of indels may be underestimated for very low sequence divergence.

2. We design the distribution such that it is time-reversible. This makes the assumptions that the probability of insertions is equal to the frequency of deletions and that these have equal length distributions. Data from DNA genome level comparisons [15,16] indicate these assumption are not necessarily true, but the effects of this on the long range evolution in proteins is not clear. The Qian-Goldstein and Benner distributions assume time reversibility since the direction of events was not known for the protein sequences they analyzed. Time-reversibility is a desirable mathematical property that is often used in sequence analysis programs for alignment and phylogeny.

3. We assume that the observed indel length distribution keeps its original form as a sum of four exponential terms at any fixed time point, and not just for time *t *= *c*. This is consistent with the assumption in the original Qian-Goldstein distribution, which fits four exponential terms. Using a function of this form allows us to scale the exponential in each term separately.

4. There are still many ways to introduce the time parameter *t *into the function. Our third assumption was then to chose a simple linear scaling of the exponents of the function with time. We found this scaling to give reasonable results when we compare the Benner distributions which were obtained at shorter time scales (see below).

With these assumptions, we then define the scaled *QG*' function for *n *> 0 as

QG'(n,t,c)=1.027×10−2e−nc/0.96t                            +  3.031×10−3e−nc/3.13t                            +  6.141×10−4e−nc/14.3t     (2)                            +  2.090×10−5e−nc/81.7t.
 MathType@MTEF@5@5@+=feaafeart1ev1aaatCvAUfKttLearuWrP9MDH5MBPbIqV92AaeXatLxBI9gBaebbnrfifHhDYfgasaacH8akY=wiFfYdH8Gipec8Eeeu0xXdbba9frFj0=OqFfea0dXdd9vqai=hGuQ8kuc9pgc9s8qqaq=dirpe0xb9q8qiLsFr0=vr0=vr0dc8meaabaqaciGacaGaaeqabaqabeGadaaakqaabeqaaiabdgfarjabdEeahjabcEcaNiabcIcaOiabd6gaUjabcYcaSiabdsha0jabcYcaSiabdogaJjabcMcaPiabg2da9iabigdaXiabc6caUiabicdaWiabikdaYiabiEda3iabgEna0kabigdaXiabicdaWmaaCaaaleqabaGaeyOeI0IaeGOmaidaaOGaemyzau2aaWbaaSqabeaacqGHsislcqWGUbGBcqWGJbWycqGGVaWlcqaIWaamcqGGUaGlcqaI5aqocqaI2aGncqWG0baDaaaakeaacaaMc8UaaGPaVlaaykW7caaMc8UaaGPaVlaaykW7caaMc8UaaGPaVlaaykW7caaMc8UaaGPaVlaaykW7caaMc8UaaGPaVlaaykW7caaMc8UaaGPaVlaaykW7caaMc8UaaGPaVlaaykW7caaMc8UaaGPaVlaaykW7caaMc8UaaGPaVlaaykW7caaMc8Uaey4kaSIaaGPaVlaaykW7cqaIZaWmcqGGUaGlcqaIWaamcqaIZaWmcqaIXaqmcqGHxdaTcqaIXaqmcqaIWaamdaahaaWcbeqaaiabgkHiTiabiodaZaaakiabdwgaLnaaCaaaleqabaGaeyOeI0IaemOBa4Maem4yamMaei4la8IaeG4mamJaeiOla4IaeGymaeJaeG4mamJaemiDaqhaaaGcbaGaaGPaVlaaykW7caaMc8UaaGPaVlaaykW7caaMc8UaaGPaVlaaykW7caaMc8UaaGPaVlaaykW7caaMc8UaaGPaVlaaykW7caaMc8UaaGPaVlaaykW7caaMc8UaaGPaVlaaykW7caaMc8UaaGPaVlaaykW7caaMc8UaaGPaVlaaykW7caaMc8UaaGPaVlabgUcaRiaaykW7caaMc8UaeGOnayJaeiOla4IaeGymaeJaeGinaqJaeGymaeJaey41aqRaeGymaeJaeGimaaZaaWbaaSqabeaacqGHsislcqaI0aanaaGccqWGLbqzdaahaaWcbeqaaiabgkHiTiabd6gaUjabdogaJjabc+caViabigdaXiabisda0iabc6caUiabiodaZiabdsha0jaaxMaacaWLjaWaaeWaaeaacqaIYaGmaiaawIcacaGLPaaaaaaakeaacaaMc8UaaGPaVlaaykW7caaMc8UaaGPaVlaaykW7caaMc8UaaGPaVlaaykW7caaMc8UaaGPaVlaaykW7caaMc8UaaGPaVlaaykW7caaMc8UaaGPaVlaaykW7caaMc8UaaGPaVlaaykW7caaMc8UaaGPaVlaaykW7caaMc8UaaGPaVlaaykW7caaMc8Uaey4kaSIaaGPaVlaaykW7cqaIYaGmcqGGUaGlcqaIWaamcqaI5aqocqaIWaamcqGHxdaTcqaIXaqmcqaIWaamdaahaaWcbeqaaiabgkHiTiabiwda1aaakiabdwgaLnaaCaaaleqabaGaeyOeI0IaemOBa4Maem4yamMaei4la8IaeGioaGJaeGymaeJaeiOla4IaeG4naCJaemiDaqhaaOGaeiOla4caaaa@22A6@

To turn equation 2 into a probability distribution (which sums to 1), we must divide the function by the sum of all values for *n *> 0 such that that

GQGc(n,t)=QG'(n,t,c)∑n=1∞QG'(n,t,c).     (3)
 MathType@MTEF@5@5@+=feaafeart1ev1aaatCvAUfKttLearuWrP9MDH5MBPbIqV92AaeXatLxBI9gBaebbnrfifHhDYfgasaacH8akY=wiFfYdH8Gipec8Eeeu0xXdbba9frFj0=OqFfea0dXdd9vqai=hGuQ8kuc9pgc9s8qqaq=dirpe0xb9q8qiLsFr0=vr0=vr0dc8meaabaqaciGacaGaaeqabaqabeGadaaakeaacqWGhbWrcqWGrbqucqWGhbWrdaWgaaWcbaGaem4yamgabeaakiabcIcaOiabd6gaUjabcYcaSiabdsha0jabcMcaPiabg2da9maalaaabaGaemyuaeLaem4raCKaei4jaCIaeiikaGIaemOBa4MaeiilaWIaemiDaqNaeiilaWIaem4yamMaeiykaKcabaWaaabmaeaacqWGrbqucqWGhbWrcqGGNaWjcqGGOaakcqWGUbGBcqGGSaalcqWG0baDcqGGSaalcqWGJbWycqGGPaqkaSqaaiabd6gaUjabg2da9iabigdaXaqaaiabg6HiLcqdcqGHris5aaaakiabc6caUiaaxMaacaWLjaWaaeWaaeaacqaIZaWmaiaawIcacaGLPaaaaaa@58E4@

We call *GQG *the Generalized Qian-Goldstein distribution. *GQG *is a scaled version of the *QG *function that describes the probability distribution of indels of length *n *(conditional on *n *> 0) at any evolutionary time *t *and assuming an evolutionary scale factor *c*.

**Figure 1:**
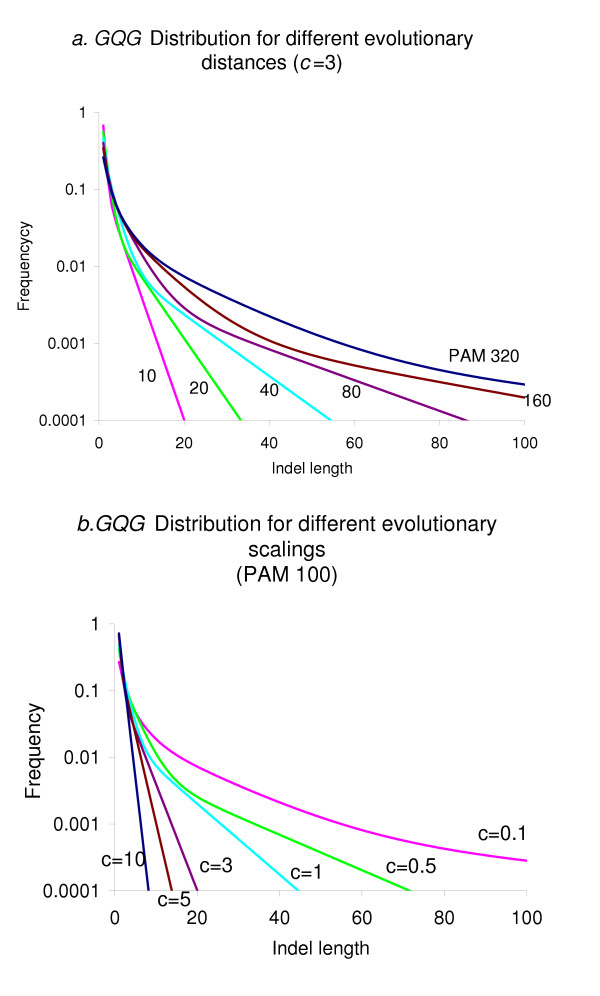
The *GCG *distribution of indel length is determined by the evolutionary distance for a given evolutionary scale factor *c*. The expected frequency of indels of given lengths are plotted. In **a**. the distribution is shown for different evolutionary distances (as labelled next to the corresponding lines). In **b**. the evolutionary distance is fixed and the *GCG *length distribution is plotted for different evolutionary scale factor values (as labelled next to the corresponding lines).

**Figure 2:**
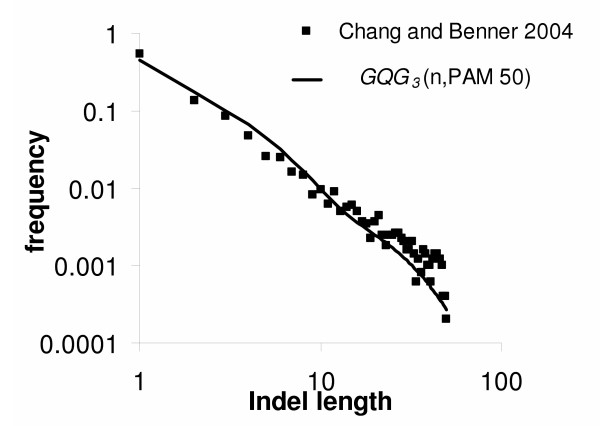
Comparison of the GQG distribution with the data obtained from the study [7] for protein sequences with less than 100 PAM sequence divergence. The parameters of the GQG disribution are set to the default *c *= 3 and *t *= PAM 50. These values were chosen simply because they seemed reasonable, not to maximize the fit of the curve to the data. The striking fit indicates that our scaling of the QG distribution is appropriate to model indels at lower levels of sequence divergence.

In Figure 1 the distribution of indel lengths is shown plotted for varying values of *c *and *t*. In figure 2, we compare the GQG distribution (with parameters *c *= 3 and PAM 50, which are very appropriate) with the data from [7] which was obtained from sequence comparisons of PAM < 100. The striking fit of the GQG distribution to data of much lower sequence divergence indicates that our scaling of the original QG distribution is appropriate.

Once defined in this way, the *GQG *does not give the frequency of indels (only their length distribution). The rates for the assumed four independent poisson processes for the appearance of indels can be combined into a single instantaneous rate *z*. The frequency of indels defines *p *such that

*p *= 1 - *e*^-*zt/c*^.     (4)

**Figure 3:**
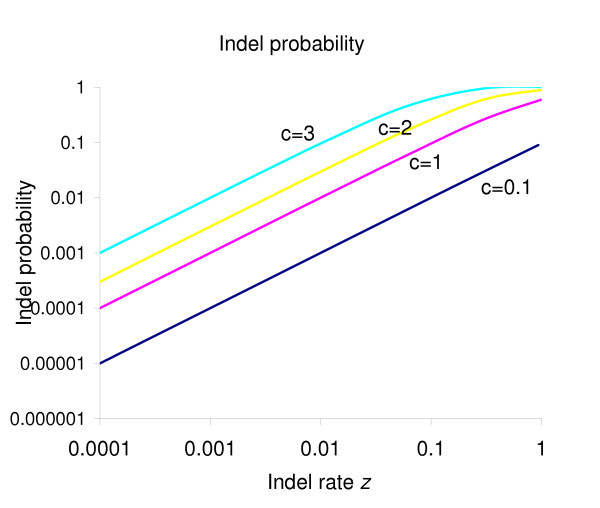
The indel probability of the *GCG *distribution is determined by the indel rate *z *(*x*-axis) and the evolutionary scale factor *c *(labelled next to the corresponding line). This probability can be set by the user to influence the number of indels present in the final alignment.

We define the indel frequency rate *p *as a parameter from which *z *can be calculated. Figure 3 shows the frequency of indels as *z *is increased for different values of the parameter *c *such that *p *= 0.03 (the observed Qian-Goldstein frequency).

By introducing parameters in the distribution, we allow a large amount of flexibility in the generation of indels and on their lengths. The indel frequency parameter *p *can modify the indel *frequency *and the evolutionary scale factor *c *parameter can be used to independently modify the distribution of indel *lengths*. Larger values of *p *will yield more indels and smaller values of will yield fewer indels. Larger values of *c *will yield shorter indels and smaller values of *c *will yield larger indels.

The original impetus for the estimation of the indel distribution was to derive gap insertion (*γ*_*I*_) and gap extension (*γ*_*E*_) penalties for use in alignment programs [1]. We used the formulas from [1] to derive an approximation for the natural log odds penalties for gaps

γE≈GQGc(3,t)−GQGc(1,t)2GQGc(2,t)     (5)
 MathType@MTEF@5@5@+=feaafeart1ev1aaatCvAUfKttLearuWrP9MDH5MBPbIqV92AaeXatLxBI9gBaebbnrfifHhDYfgasaacH8akY=wiFfYdH8Gipec8Eeeu0xXdbba9frFj0=OqFfea0dXdd9vqai=hGuQ8kuc9pgc9s8qqaq=dirpe0xb9q8qiLsFr0=vr0=vr0dc8meaabaqaciGacaGaaeqabaqabeGadaaakeaacqaHZoWzdaWgaaWcbaGaemyraueabeaakiabgIKi7oaalaaabaGaem4raCKaemyuaeLaem4raC0aaSbaaSqaaiabdogaJbqabaGccqGGOaakcqaIZaWmcqGGSaalcqWG0baDcqGGPaqkcqGHsislcqWGhbWrcqWGrbqucqWGhbWrdaWgaaWcbaGaem4yamgabeaakiabcIcaOiabigdaXiabcYcaSiabdsha0jabcMcaPaqaaiabikdaYiabdEeahjabdgfarjabdEeahnaaBaaaleaacqWGJbWyaeqaaOGaeiikaGIaeGOmaiJaeiilaWIaemiDaqNaeiykaKcaaiaaxMaacaWLjaWaaeWaaeaacqaI1aqnaiaawIcacaGLPaaaaaa@547B@

γI≈log⁡(p1−eγE)+2γE.     (6)
 MathType@MTEF@5@5@+=feaafeart1ev1aaatCvAUfKttLearuWrP9MDH5MBPbIqV92AaeXatLxBI9gBaebbnrfifHhDYfgasaacH8akY=wiFfYdH8Gipec8Eeeu0xXdbba9frFj0=OqFfea0dXdd9vqai=hGuQ8kuc9pgc9s8qqaq=dirpe0xb9q8qiLsFr0=vr0=vr0dc8meaabaqaciGacaGaaeqabaqabeGadaaakeaacqaHZoWzdaWgaaWcbaGaemysaKeabeaakiabgIKi7kGbcYgaSjabc+gaVjabcEgaNnaabmaabaWaaSaaaeaacqWGWbaCaeaacqaIXaqmcqGHsislcqWGLbqzdaahaaWcbeqaaiabeo7aNnaaBaaameaacqWGfbqraeqaaaaaaaaakiaawIcacaGLPaaacqGHRaWkcqaIYaGmdaWgaaWcbaGaeq4SdC2aaSbaaWqaaiabdweafbqabaaaleqaaOGaeiOla4IaaCzcaiaaxMaadaqadaqaaiabiAda2aGaayjkaiaawMcaaaaa@486B@

## Implementation

We have implemented the Generalized Qian-Goldstein distribution in a program called Simprot to simulate protein sequence evolution. Given a bifurcating phylogenetic tree, children sequences inherit the sequence of their parent with modification due to mutation events. The number of mutations expected depends on the length of evolutionary time that separates the child from the parent and their type is determined by the chosen models. Substitutions are made according to the user-chosen substitution model. Insertions and deletions are made according to the *GQG *model described above. The user determines the values of the evolutionary scale factor *c *which controls the indel length distribution, and the indel frequency rate *p *which determines their frequencies. The shape parameter for the gamma model of [13] distribution of evolutionary rates is also determined by the user.

The parameters for the models are input via an interface screen (available through the Web, or by download for local Windows and Unix/Linux systems). The locally installed versions allow several input screens such that the resulting simulated protein sequences will consist of segments each evolving according to its own set of parameters and tree.

The program generates sequences according to the chosen indel and substitution models and outputs the alignments of sequences from the terminal branches. When several protein segments have been selected, the sequences are appropriately fused into single sequences by matching the names of the terminal taxons in the input tree files. The gap opening and gap extension penalties corresponding to the input parameters and time *t *= *c *for each protein segment is an additional output provided by the program for user reference.

### Evolution

Each protein segment is simulated independently. Simprot parses the given tree file into a tree structure to use as a guide in simulating evolution. It then generates a random amino acid sequence of given length *r *at the root of the tree according to the equilibrium frequency of amino acids in the substitution model. Each amino acid site is assigned a rate of evolution based on the gamma distribution. The program then recursively generates mutations on the protein sequence at each of the tree nodes. There are two types of mutations: insertion/deletion and substitution. Indels are performed before substitutions at each tree node.

#### Number of indels

Simprot assumes a Poisson process for insertion and deletions and thus the expected frequency of indels (of any length) in a sequence is

*p *= 1 - *e*^-*zt/c*^,     (7)

where *z *is the indel probability and *t *is the branch length to the daughter sequence which is scaled by the evolutionary scale factor. For each amino acid site, a uniformly distributed random number is picked to check whether it is lower than the expected frequency. The number of times this happens over the entire sequence becomes the number of indels that will be performed.

#### Indel positions

Indel sites are chosen according to their rate of evolution as given by the gamma distribution. This means that sites more likely to substitute will also be more likely to have an insertion or deletion.

#### Indel length

To determine the length of an indel after choosing to create one, the cumulative distribution function (CDF) of the indel-length probabilities for *n *> 0 as determined by the *GQG *model is evaluated using Eq. 3. A cap on the indel length is also applied. Indels must be shorter than the maximum indel length or 5% of the sequence length (whichever is smaller).

#### Indel type

Simprot chooses between insertion and deletion with equal probability. If insertion is chosen, an amino acid sequence of the indel length is generated (according to the same amino acid frequency distribution that generated the root sequence) and inserted before the indel position. If deletion is chosen, the indel length of amino acids are deleted beginning with the current position. If the length of the indel is greater than the number of amino acids in the sequence following the current position, additional amino acids are deleted towards the start of the sequence.

The probability of amino acids being inserted and deleted is the same so that the length of the sequences should remain approximately the same. The sequence is updated after each indel event and all indels are performed before substitutions.

#### Substitutions

Once all indels have been performed at a given node, Simprot performs substitutions of the individual amino acid according to the evolutionary substitution model. Currently the models implemented are PAM, JTT and PMB. The substitution probabilities are calculated from the previously calculated eigenvalue decomposition of the probability matrix. This strategy, first used by Felsenstein in the Phylip package [17] facilitates computation of the substitution probabilities for any branch length. The model considers the probability of all amino acid substitutions for a given branch length times the evolutionary rate at the site (as determined by the gamma model). As the program traverses the tree, the descendant nodes inherit the mutations generated.

### Alignment

A copy of the "true" sequence alignment is also produced for the generated sequence family. At each node, the locations of insertions and deletions are maintained relative to the sequence at the parent node. This correspondence is called the "gapped sequence" because gap characters (-) are inserted in copies of both the current sequence and the parent sequence to represent the correspondence. After the sequence family has been generated, a recursive traversal rebuilds the true alignment using the gapped sequences. The procedure makes use of the fact that, for any node in the tree, the true alignments are known for the sequences in the left and right subtrees from this node, and the gapped sequences can be used to align these two true alignments, producing the true alignment for all sequences below the root. This procedure requires only a linear traversal of the tree, and therefore imposes no significant additional cost of computation. Simprot outputs the aligned sequences from the leaves of the tree in Fasta and Phylip format. It also creates a file of the set of unaligned protein sequences. If the protein is segmented, the files for the segments are merged into the final alignment.

## Conclusion

While the process of amino acid substitution has been extensively studied and modelled, there has been relatively little study of the insertion-deletion process in protein coding sequences [18]. The model we propose may not fit all proteins but it has the properties of being based on an empirically derived distribution, and being flexible so as to allow a user to test many conditions. We plan to use additional empirical data of the frequency and distribution of indels in proteins to refine our model in subsequent releases of Simprot. The alignments generated by Simprot will be useful for testing methods of analysis of protein sequence families. It will be particularly useful for the development of new alignment methods, phylogenetic tree building, detection of recombination and horizontal gene transfer, and homology detection, where knowing the true course of sequence evolution is essential.

## Availability and requirements

Project name: Simprot

Project home page: 

Operating systems: Linux, Windows 95 or later (local installation)

Programming language: C++

License: University of Illinois/NCSA Open Source License

Any restrictions to use by non-academics: no

## List of abbreviations

PMB probability matrix from Blocks, JTT Jones Taylor Thorton, PAM Percent Accepted Mutation, GQG Generalized Qian-Goldstein distribution, CDF cumulative distribution function.

## Authors' contributions

AP implemented the GQG distribution in Simprot and helped draft the manuscript. ADS implemented the indel and substitution processes in Simprot and helped draft the manuscript. PASN implemented the gap penalties, created the GUI interfaces and provided comments on the manuscript. ERMT derived the GQG distribution, supervised the project and approved the final manuscript.
